# Can docetaxel combined prednisone effectively treat hormone refractory prostate cancer? A protocol of systematic review and meta-analysis

**DOI:** 10.1097/MD.0000000000020367

**Published:** 2020-05-29

**Authors:** Liang Cheng, Cai-Fang Yue, Yue Wang, Hui-Feng Cao, Jian-Feng Dong, Long-Xin Dong, Da-Yin Chen

**Affiliations:** a Department of Urology; b Department of Critical Care Medicine, The First Affiliated Hospital of Jiamusi University; c Department of Pathology, Jiamusi Anorectal Hospital, Jiamusi, China.

**Keywords:** docetaxel, efficacy, hormone refractory prostate cancer, prednisone, safety

## Abstract

**Background:**

Previous studies have reported that docetaxel combined prednisone (DP) has been used for the treatment of patients with hormone refractory prostate cancer (HRPC). However, its results are still inconsistent. Therefore, this study will synthesize the latest evidence of the efficacy and safety of DP for the treatment of patients with HRPC.

**Methods:**

Cochrane Library, PUBMED, EMBASE, Web of Science, CINAHL, CBM, and CNKI will be searched to identify randomized controlled trials published from their inception to the March 1, 2020, irrespective language and publication time restrictions. We will calculate the pooled effects of dichotomous outcomes as risk ratio and 95% confidence intervals, and that of continuous outcomes as standardized mean difference or mean difference and 95% confidence intervals. Study quality will be assessed using Cochrane risk of bias, and quality of evidence for main outcome will be evaluated using Grading of Recommendations Assessment Development and Evaluation. Statistical analysis will be performed using RevMan 5.3 software.

**Results:**

This study will appraise the efficacy and safety of DP for the treatment of patients with HRPC. The primary outcome includes overall survival, and the secondary outcomes comprise of progression-free survival, prostate-specific antigen response rate, duration of prostate-specific antigen response, objective tumor response rate, disease-free survival, quality of life, and adverse events.

**Conclusion:**

The results of this study may provide helpful evidence of DP for the treatment of patients with HRPC.

Systematic review registration: INPLASY202040112.

## 1 Introduction

Prostate cancer is the most common cancer in males around the world.^[1–4]^ It accounts for 14% of new cancers and 6% of cancer deaths in male patients.^[5–6]^ Previous studies found that prostate cancer is an androgen-dependent disease, and androgen deprivation therapy (also known as hormone therapy) has been used for its management.^[7–11]^ However, many patients develop to hormone refractory prostate cancer (HRPC).^[12–15]^ Although several studies have reported the efficacy and safety of docetaxel combined prednisone (DP) for the treatment of HRPC,^[16–26]^ no systematic review assessed efficacy and safety of DP for the management of HRPC. Therefore, this study will systematically assess the efficacy and safety of DP for the treatment of HRPC.

## 2 Objective

The objective of this study is to systematically investigate the efficacy and safety of DP for the treatment of patients with HRPC.

## 3 Methods

### 3.1 Study registration

This study has been registered on INPLASY202040112. It has been conducted according to the Preferred Reporting Items for Systematic Reviews and Meta-Analysis Protocol statement guidelines.^[27]^

### 3.2 Dissemination and ethics

We plan to publish this study on an academic journal or a conference meeting. This study will not need ethical approval, because no privacy data will be collected.

### 3.3 Inclusion criteria

#### 3.3.1 Participants

We will consider all male patients (aged 18 years or older) who were diagnosed as HRPC regardless their country, race, and severity of HRPC.

#### 3.3.2 Interventions/exposure

Patients in the experimental group received DP treatment alone.

Patients in the control group were allowed to receive all treatments, such radiotherapy, and surgery. However, we will exclude patients who also underwent any forms of DP.

#### 3.3.3 Study types

We will include studies designed as all randomized controlled trials (RCTs) that specifically explore the efficacy and safety of DP for the treatment of patients with HRPC. We will exclude any other unqualified studies, such as laboratory studies, case report, case series, review, and non-clinical trials.

#### 3.3.4 Outcome measurements

Primary outcome is overall survival. Secondary outcomes are progression-free survival, prostate-specific antigen response rate, duration of prostate-specific antigen PSA response, objective tumor response rate, disease-free survival, quality of life (as measured by any relevant scales reported in the trials), and adverse events.

#### 3.3.5 Literature search

The following electronic databases of Cochrane Library, PUBMED, EMBASE, Web of Science, CINAHL, CBM, and CNKI will be examined to identify RCTs published from their inception to the March 1, 2020, regardless language and publication time limitations. All RCTs that test the efficacy and safety of DP for the treatment of patients with HRPC will be included in this study. The search strategy of PUBMED is presented in Table 1. We will also adapt similar search strategies for other electronic databases.

**Table 1:**
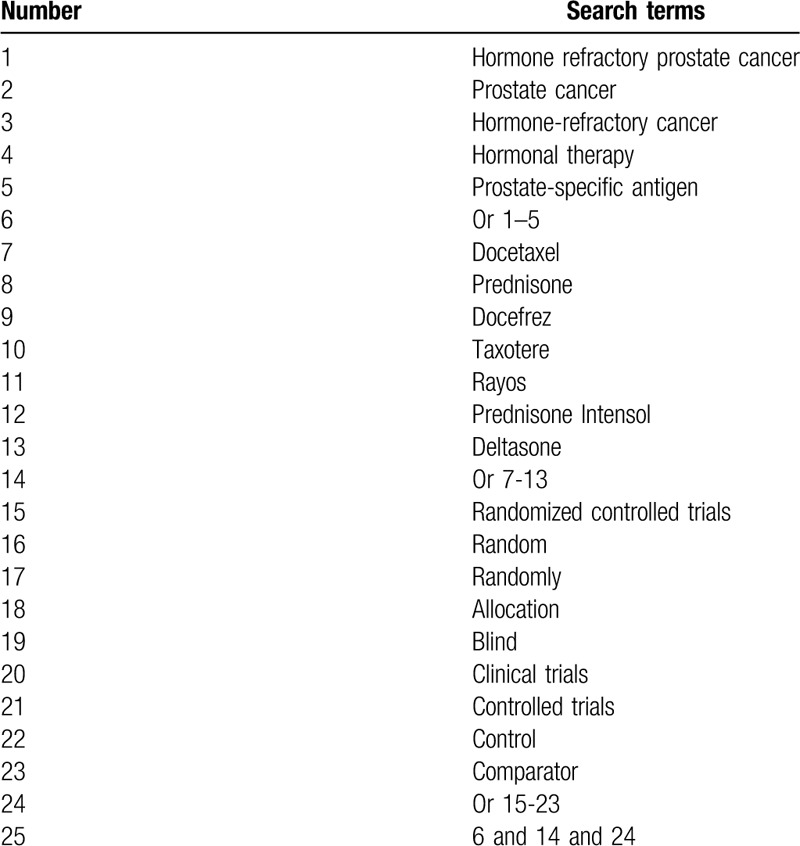
Search strategy sample used in PUBMED.

In addition, we will also check other literature sources, such as dissertations, conference proceedings, and reference lists of included trials.

### 3.4 Study selection

Two investigators will independently examine the titles/abstracts of all searched records and any studies that are not related to the topic will be eliminated. Then, full-text of the remaining studies will be obtained and will be carefully identified against all inclusion criteria. Any divergences will be solved by a third investigator through discussion. A flow diagram of study selection will be presented.

### 3.5 Data extraction and management

Two investigators will independently extract data based on the pre-designed data extraction form. It includes study information (eg, title, first author, and publication year), patient characteristics (eg, age, race, sample size, eligibility criteria, and duration and severity of HRPC), trial setting, study design (eg, details of randomization, allocation, and blind), interventions and controls (eg, types of modalities, dosage, and session), outcomes, results, findings, and other relevant information. Any differences will be solved by a third investigator through discussion. Any missing data will be obtained from primary authors. If it is not available, we will only analyze data at hand using intention-to-treat analysis.

### 3.6 Study quality assessment

Two investigators will independently appraise study quality of each eligible trial using Cochrane Collaboration's tool. Each trial will be assessed through seven aspects, and each item is rated as high, unclear or low risk of bias. Any discrepancies will be solved by a third investigator through consultation.

### 3.7 Measurement of treatment effect

The pooled effects of continuous data will be expressed as mean difference or standardized mean difference and 95% confidence intervals. The pooled effects of dichotomous data will be exerted as risk ratio and 95% confidence intervals.

### 3.8 Assessment of heterogeneity

We will utilize *I*^*2*^ test to estimate statistical heterogeneity. It will be defined *I*^*2*^ ≤ 50% as acceptable heterogeneity, and *I*^*2*^ > 50% as significant heterogeneity.

### 3.9 Data synthesis

RevMan 5.3 software will be employed for data synthesis and meta-analysis if it is possible. We will use a fixed-effect model to pool the outcome data if acceptable heterogeneity is examined, and meta-analysis will be performed if sufficient data is collected. Otherwise, we will apply a random-effect model to synthesize the outcome data if considerable heterogeneity is found, and subgroup analysis will be carried out. If a meta-analysis cannot be conducted, we will perform a narrative summary to elaborate outcome results.

### 3.10 Subgroup analysis

We will carry out a subgroup analysis to figure out sources of obvious heterogeneity across studies according to the different types of treatments, controls, and outcomes.

### 3.11 Sensitivity analysis

We will conduct a sensitivity analysis to test the robustness of study findings by removing studies with high risk of bias.

### 3.12 Reporting bias

We will undertake a funnel plot and Egger regression test to identify any possible reporting biases if at least 10 RCTs are included.

## 4 Discussion

There have been RCTs suggesting that administration of DP may be a promising medication for the treatment of patients with HRPC.^[16–26]^ However, no systematic review has explored this topic. To our best knowledge, this study will be the first one to investigate the efficacy and safety of DP for patients with HRPC. It will be performed according to the comprehensive literature search from electronic databases and grey literatures. With all eligible RCTs, the results of this study will provide up-to-date evidence on the efficacy and safety of DP in treatment of patients with HRPC.

## Author contributions

**Conceptualization:** Liang Cheng, Cai-Fang Yue, Yue Wang, Hui-Feng Cao, Long-Xin Dong, Da-Yin Chen.

**Data curation:** Liang Cheng, Yue Wang, Hui-Feng Cao, Jian-Feng Dong, Long-Xin Dong, Da-Yin Chen.

**Formal analysis:** Cai-Fang Yue, Yue Wang, Hui-Feng Cao, Da-Yin Chen.

**Funding acquisition:** Da-Yin Chen.

**Investigation:** Da-Yin Chen.

**Methodology:** Liang Cheng, Cai-Fang Yue, Hui-Feng Cao, Jian-Feng Dong, Long-Xin Dong.

**Project administration:** Da-Yin Chen.

**Resources:** Liang Cheng, Cai-Fang Yue, Yue Wang, Hui-Feng Cao, Jian-Feng Dong, Long-Xin Dong.

**Software:** Liang Cheng, Cai-Fang Yue, Hui-Feng Cao, Jian-Feng Dong, Long-Xin Dong.

**Supervision:** Da-Yin Chen.

**Validation:** Liang Cheng, Yue Wang, Hui-Feng Cao, Long-Xin Dong, Da-Yin Chen.

**Visualization:** Liang Cheng, Cai-Fang Yue, Jian-Feng Dong, Da-Yin Chen.

**Writing – original draft:** Liang Cheng, Cai-Fang Yue, Hui-Feng Cao, Da-Yin Chen.

**Writing – review & editing:** Liang Cheng, Cai-Fang Yue, Yue Wang, Jian-Feng Dong, Long-Xin Dong, Da-Yin Chen.
